# Revealing the quantum nature of the voltage-induced conductance changes in oxygen engineered yttrium oxide-based RRAM devices

**DOI:** 10.1038/s41598-023-49924-2

**Published:** 2024-01-11

**Authors:** F. L. Aguirre, E. Piros, N. Kaiser, T. Vogel, S. Petzold, J. Gehrunger, C. Hochberger, T. Oster, K. Hofmann, J. Suñé, E. Miranda, L. Alff

**Affiliations:** 1 Departament d’Enginyeria Electrònica, Universitat Autònoma de Barcelona, 08193 Cerdanyola del Valles, Spain; 2 Intrinsic Semiconductor Technologies, Ltd., Buckinghamshire, United Kingdom; 3 Advanced Thin Film Technology Division, Institute of Materials Science, Technische Universität Darmstadt, Darmstadt, Germany; 4 Computer Systems Group, Department of Electrical Engineering and Information Technology, Technische Universität Darmstadt, Darmstadt, Germany; 5 Integrated Electronic Systems Lab, Department of Electrical Engineering and Information Technology, Technische Universität Darmstadt, Darmstadt, Germany

**Keywords:** Materials for devices, Electronic devices

## Abstract

In this work, the quasi-analog to discrete transition occurring in the current–voltage characteristic of oxygen engineered yttrium oxide-based resistive random-access memory (RRAM) devices is investigated in detail. In particular, the focus of our research is not on the absolute conductance values of this characteristic but on the magnitude of its conductance changes occurring during the reset process of the device. It is found that the detected changes correspond to conductance values predominantly of the order of the quantum unit of conductance *G*_*0*_ = 2*e*^2^/*h*, where *e* is the electron charge and *h* the Planck constant. This feature is observed even at conductance levels far above *G*_*0*_, i.e. where electron transport is seemingly diffusive. It is also observed that such behavior is reproducible across devices comprising yttrium oxide layers with different oxygen concentrations and measured under different voltage sweep rates. While the oxygen deficiency affects the total number of quantized conductance states, the magnitude of the changes in conductance, close to 1 *G*_*0*_, is invariant to the oxygen content of the functional layer.

## Introduction

Resistive random-access memory (RRAM) is one of the most promising emerging non-volatile memory technologies as it is characterized by competitive performance such as high scalability (< 10 nm)^1^, ultrafast (< 1 ns)^2^ and low-power switching (1 pJ per bit)^3^, high endurance (> 10^9^ cycles)^4^ and good radiation hardness^5,6^. RRAM devices rely on the resistive switching mechanism which has been observed in a number of transition metal oxides such as hafnium^7^, tantalum^8^, and yttrium^9^ oxide, all of which are considered as alternative gate oxide materials in complementary metal–oxide–semiconductor (CMOS) technology. As it has been reported several times, in valence change memories, the information storage relies on the formation and rupture of conductive filaments formed of oxygen vacancies^7^. When the vacancy bridge connects both electrodes, the device switches to the low resistive state (LRS), whereas when the filament is ruptured at opposite bias, the high resistive state (HRS) is recovered. According to mesoscopic theory^10^, when the cross-section of the conductive filament is comparable to the electronic wavelength, quantum effects are expected to arise in the system even at room temperature. Under this circumstance, the constriction’s bottleneck behaves as a quantum point contact (QPC) in which the electron transport exhibits quantized steps in terms of the fundamental unit of conductance (quantum conductance, *G*_*0*_ = *2e*^2^*/h* = (12.9 kΩ)^−1^ ^11–13^, where *e* is the electron charge and *h* the Planck constant). The stabilization of a large number of quantized conductance steps can be potentially utilized for (quantized) multi-bit storage ^14,15^. The resulting quasi-analog to discrete switching is also desired for neuromorphic applications that mimic the synaptic connectivity of biological neurons^16^.

In the last years, yttrium oxide (Y_2_O_3_) has been gaining increasing interest as the active material in filamentary-type RRAMs because of the intrinsically vacant anion sublattice sites occurring even in the stoichiometric compound, resulting in a universal low-frequency noise and good thermal stability^16–19^. The intrinsic oxygen vacancies are reported to form chains along the [110] directions within the cubic phase that can serve as preferential paths for the filament formation, thus contributing to the low electroforming and operation voltages^9,20,21^. It has also been shown^16^ that by engineering the oxygen content, a digital-to-(quasi-)analog transition can be induced in the switching characteristics: by increasing the degree of oxygen deficiency a high number of stable intermediate resistive states are created during both the set and reset processes. Moreover, these intermediate levels can be described in terms of integer and non-integer values of *G*_*0*_, i.e., they display nonlinear conductance quantization as understood in the framework of the generalized mesoscopic transport model^16^. In this work, we do not focus on the quantized conductance levels themselves, but specifically on the magnitude of the transitions between intermediate states occurring during the reset process. The important result is that oxygen deficient Y_2_O_x_ not only presents a multi-step or even fully analog/gradual switching, but that the transition from one level to the next occurs mostly in steps of ~ 1 *G*_*0*_. This phenomenon occurs regardless of the oxygen content in the oxide layer, as the stoichiometry of the functional layer affects mostly the total number of transitions observed but not their magnitude. This indicates that the observed quantum behavior of the electron transport is a universal property in filamentary structures revealed when considering conductance *changes* instead of a*bsolute values*.

## Results and discussion

The experiments shown in this work were conducted on RRAM devices consisting of a Si/Al/TiN/Y_2_O_3−x_/Pt structure. The functional layer was deposited using a molecular beam epitaxy setup that allows for controlling the oxygen stoichiometry of the yttria film by varying the flow of oxygen radicals (in total 5 different samples grown with -from least to most oxidized- 0.1, 0.2, 0.3, 0.5, and 1 standard cubic centimeter per minute, sccm) and the film growth rate (1 angstrom per second, Å s^−1^, for all samples except for the most oxidized one grown with 0.25 Å s^−1^). The electrode area is 30 × 30 µm^2^ and the oxide thickness is 18.9 nm for the 0.1 sccm case, 16.8 nm for 0.2 sccm, 15.9 nm for 0.3 sccm, 15 nm for 0.5 sccm and 13.2 nm for 1 sccm. In all cases the exact thickness values have been derived from the period of the measured X-ray reflectometry (XRR) oscillations (using an RCRefSim software ^22^). The yttria layers grown with equal to or higher than 0.3 sccm oxygen flow are characterized by a mixture between the monoclinic and cubic phases, whereas for the most oxygen deficient films (0.1 and 0.2 sccm) only a cubic phase is observed. All yttrium oxide layers are polycrystalline. The fabrication process and the complete structural characterization of the samples were described elsewhere ^16^. The electrical characterization was carried out using a Keithley 4200 semiconductor characterization system (SCS), biasing the Pt top electrode and grounding the TiN bottom electrode. The internal current compliance of the SCS was used in order to avoid the hard breakdown of the oxide layer during electroforming and set operations.

**Figure 1:**
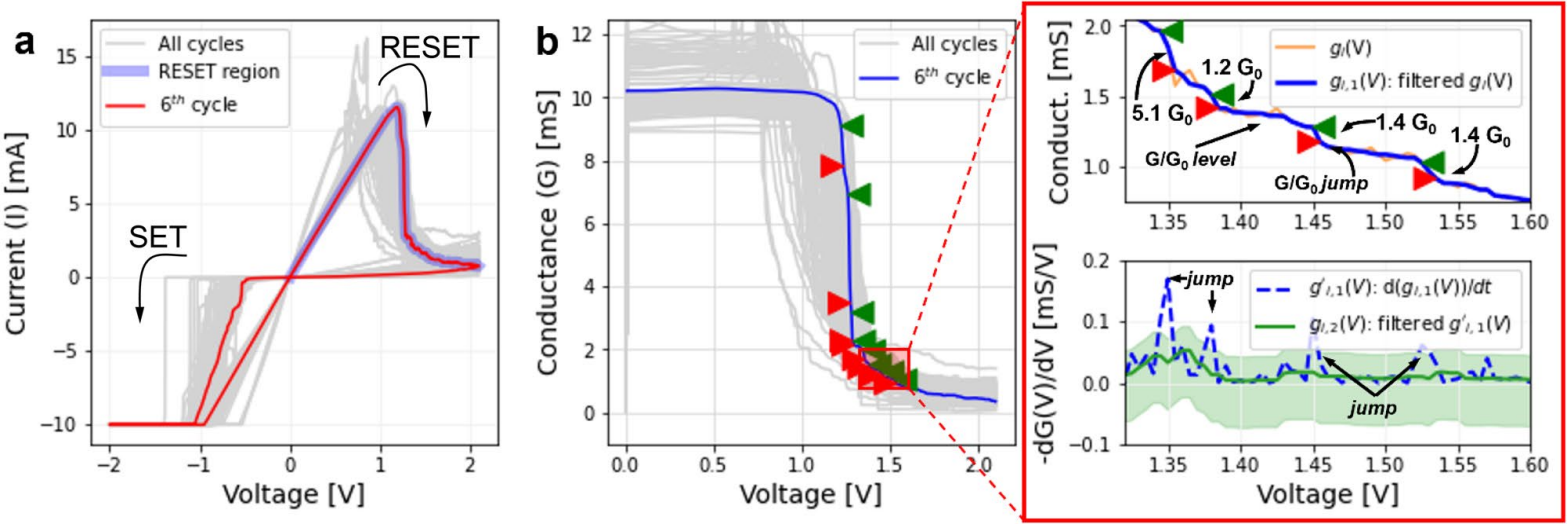
(**a**) *I–V* loops corresponding to the least oxidized sample (0.1 sccm oxygen flow during growth). The region (reset transition) used to extract the information about the transitions is highlighted in blue. (**b**) *G-V* representation of the reset transition. The green and red markers identify the starting and ending point of each jump. The zoom-in-panel at the right side of figure (**b**) shows a detail of the identified jumps (top) and how they are assessed using the derivative of the signal (bottom).

As previously reported^16^, oxygen engineering in yttria films allows to control the density of oxygen vacancies in the functional layer, which in turn enables the control over the dynamics of the filament formation and rupture. In highly oxidized yttria grown with 1 sccm oxygen flow, the switching is characterized by an abrupt transition. By decreasing the quantity of oxygen in the film, this behavior can be transitioned to a multilevel (at moderate oxidation, 0.5 sccm) and eventually, with further reduction of the oxygen content (below 0.5 sccm), to a quasi-analog switching characteristic both for the set and reset processes. This is illustrated in Fig. 1a.

To statistically study the distribution of the conductance variation in terms of *G*_*0*_, the reset transition was analyzed for 100 successive current–voltage (*I–V*) loops (see Fig. 1a). The obtained results are in agreement with previous works on this subject^12,15,23–27^. Data from the reset region were converted into conductance-voltage (*G–V*, further referred as ${g}_{i}(v)$ with *i* identifying the loop number) and post-processed with a moving median filter^28^ ${\mathcal{F}}_{MM}$ to obtain a signal ${g}_{i,1}\left(v\right)={\mathcal{F}}_{MM}\left\{{g}_{i,1}(v)\right\}$ free of noise but preserving the sharp edges corresponding to the transitions from one conductance level to another. The resulting ${g}_{i,1}\left(v\right)$ curve is presented in Fig. 1b, and its derivative computed $\left({g{\prime}}_{i,1}\left(v\right)=\frac{d({g}_{i,1}(v))}{dv}\right)$. By further filtering ${g{\prime}}_{i,1}\left(v\right)$ using a moving average filter^28^ ${\mathcal{F}}_{MA}$ to obtain a reference signal ${g}_{i,2}\left(v\right)={\mathcal{F}}_{MA}\left\{{g{\prime}}_{i,1}\left(v\right)\right\}$, it is possible to systematically identify the transitions between two different conductance levels by comparing ${g{\prime}}_{i,1}\left(v\right)$ against ${g}_{i,2}\left(v\right)+\delta$, $\delta$ being a threshold defining the minimal acceptable value of a conductance transition to be recognized as such, which in this case is 0.2 *G*_*0*_ (see the green shaded region around ${g}_{i,2}\left(v\right)$ in Fig. 1b). The zoomed region in Fig. 1b exemplifies the procedure by presenting in the top axis a superposition of the signals ${g{\prime}}_{i,1}\left(v\right)$ and ${g}_{i,2}\left(v\right)$ with the tolerance region defined by ${g}_{i,2}\left(v\right)+\delta$. The transition conductance levels are identified by the points in which ${g{\prime}}_{i,1}\left(v\right)>{g}_{i,2}\left(v\right)+\delta$ and they are indicated in the bottom chart of the zoomed region by the green (start of the transition) and red (end of the transition) markers, which shows ${g}_{i,1}\left(v\right)$ for the same voltage region. Other examples are reported in Supplementary Fig. 1 to complement this assertion.

**Figure 2:**
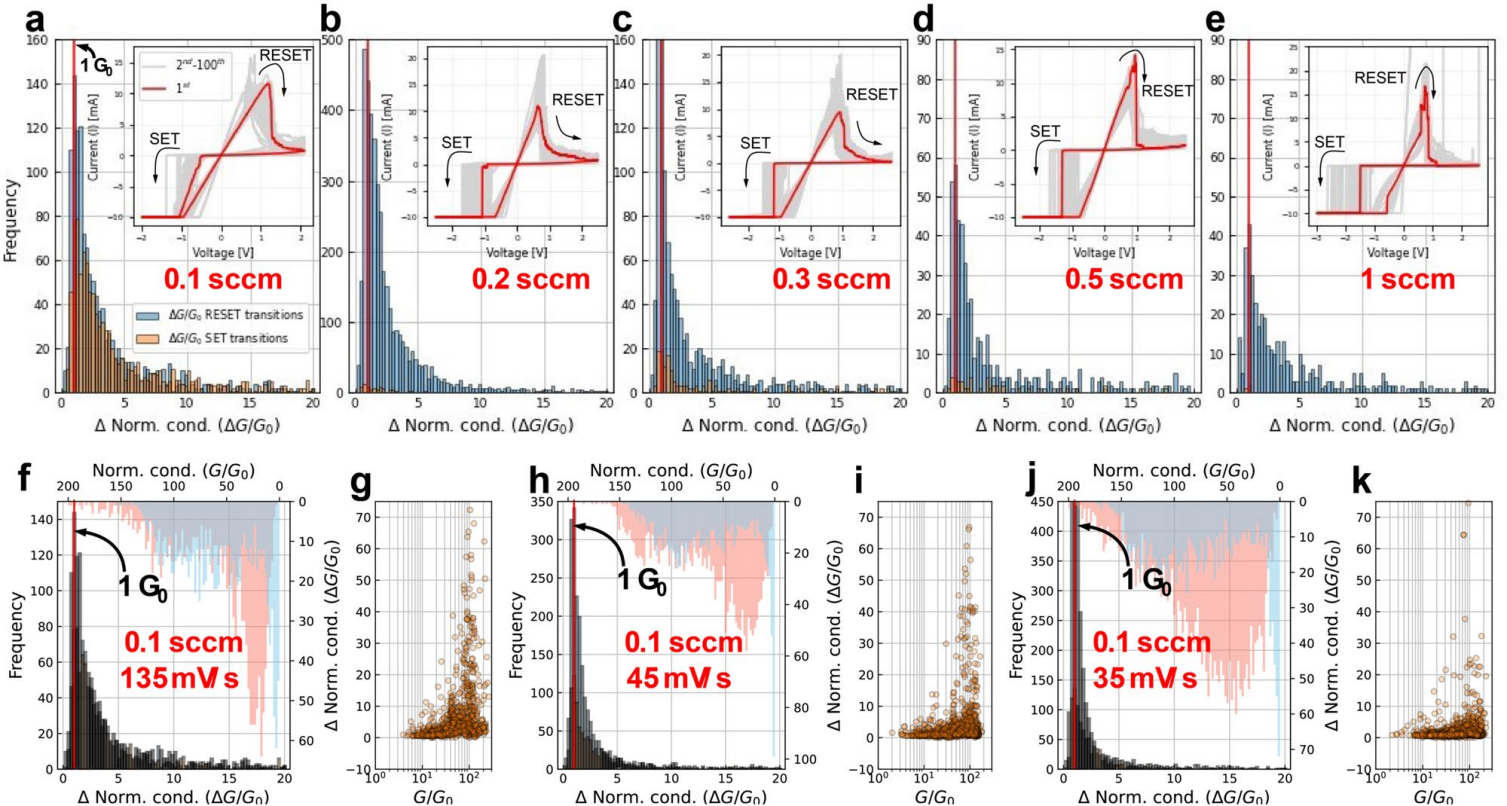
Histograms of the conductance transitions for each oxidation condition. (**a**) 0.1 sccm, (**b**) 0.2 sccm, (**c**) 0.3 sccm, (**d**) 0.5 sccm and (**e**) 1 sccm. The inset presents the overall aspect of the associated *I–V* loops. Note that very few transitions are found during the set event for oxidations larger than 0.1 sccm. This is due to the very abrupt set transition. Histograms of the conductance transitions for three different samples with the same oxidation condition but obtained with different ramp rates. (**f**) 135 mV/sec. (**h**) 45 mV/sec. (**j**) 35 mV/sec. The bottom x-axis indicates the histogram of the quantum transitions during the SET/RESET events and the upper x-axis indicates the histogram of conductance levels in units of *G*_*0*_ found during the transition. The panels at the right of each histogram reports the correlation plot between the conductance level and the conductance transitions (**g**, **i**, **k**).

The method described above was used to locate the transitions between conductance levels on the five different oxygen engineered yttria devices investigated. The resulting histograms for the normalized conductance steps ($\Delta G/{G}_{0})$ are shown in Fig. 2a–e. The insets show the *I–V* loops for the sample under test. The observed cycle-to-cycle variability is a result of morphological changes in the structure of the conducting pathway^29,30^. For oxygen flows above 0.1 sccm (Fig. 2b–e), the number of transitions between conductance levels is found to be significantly lower during the set process, which is in line with the set transitions becoming increasingly abrupt when increasing the oxygen content. A similar behavior is observed in the reset transition, in which, despite showing some conductance transitions, the total count decreases for higher oxygen flows. The key finding is that the transitions between conductance levels during the reset process mostly occur in steps of ~ 1 *G*_*0*_, this being independent of the degree of oxygen deficiency. In fact, for the case of the set process observed in the sample presented in Fig. 2a (oxygen flow of 0.1 sccm and growth rate of 1 Å s^−1^) which also shows quantized conductance states, the transitions between them mostly occur in steps of ~ 1 *G*_*0*_. To confirm the findings reported in Fig. 2a–e, the identification of the transitions between conductance levels was repeated in other two devices fabricated in the same way as the one reported in Fig. 2a but varying the voltage sweep ramp rate from ~ 135 mV/s (Fig. 2f) to ~ 45 mV/s (Fig. 2h) and to ~ 35 mV/s (Fig. 2j). When probing conductance quantization with the voltage-sweep method, it is crucial to use a voltage-step size small enough to probe quantum conductance steps with the highest possible resolution and to keep simultaneously the overall measurement time at a reasonable level^13^. The joint assessment of Fig. 2f,h,j indicates that regardless of the voltage sweep rate, the general trend remains the same, that is, transitions between successive conductance levels are in the vast majority of ~ 1 *G*_*0*_, i.e. the conductance value expected for an ideal monomode ballistic conductor. Finally, by considering the correlation plots between normalized conductance levels at which the transitions take place (*G/G*_*0*_), and the transition itself (*ΔG/G*_*0*_), it is possible to see in Fig. 2g,i,k (and in more detail in Supplementary Fig. 2) that the small transitions between quantized conductances take place mostly for conductance values close to *G*_*0*._ This behavior is reasonable since (i) the conductance state cannot reach lower conductance values unless a deep reset is performed (requiring higher voltages) with the consequent formation of a gap and (ii) conductance quantization is observable only when the lateral size of the constriction is comparable to the electron wavelength, which is not the case for highly conductive filaments^13^.

The different likelihood of the set and reset processes to exhibit conductance quantization effects as a function of the oxygen flow used for fabrication can be explained by considering the different mechanisms involved in each of these switching events^31^. In general, for oxide-based bipolar switching memristors, the set process is much faster and thus more abrupt than the reset process, which hinders the observation of quantization effects^12,32,33^. During the set transition, the movement of ions is activated by both the electric field and Joule heating related to thermal effects, with Soret forces (thermophoresis) driving oxygen vacancies towards the conducting channel under a steep temperature gradient occurring in the oxygen-rich parent phase, therefore creating a positive feedback loop between field-induced and temperature effects.^34–37^ On the other hand, during reset, the heat-assisted and concentration gradient-driven Fick diffusion dominates over thermophoresis as the oxygen-poor conducting path is characterized by higher heat and electrical conductivity than the surrounding insulating matrix. This leads to the creation of a flatter temperature gradient resulting in a slower switching dynamic. It has been shown that in low conductive filaments (i.e. when approaching the HRS regime) the contribution of phonon heat conduction allows decreasing the temperature^36^. This also means that the introduction of a higher degree of oxygen deficiency improves the electron and heat conduction in the functional layer, thus ultimately minimizing the contribution of thermal effects and making the field-driven drift of oxygen ions dominant, allowing for a higher controllability of the switching dynamics and the stabilization of intermediate resistance states both in the set and reset processes^16^.

**Figure 3:**
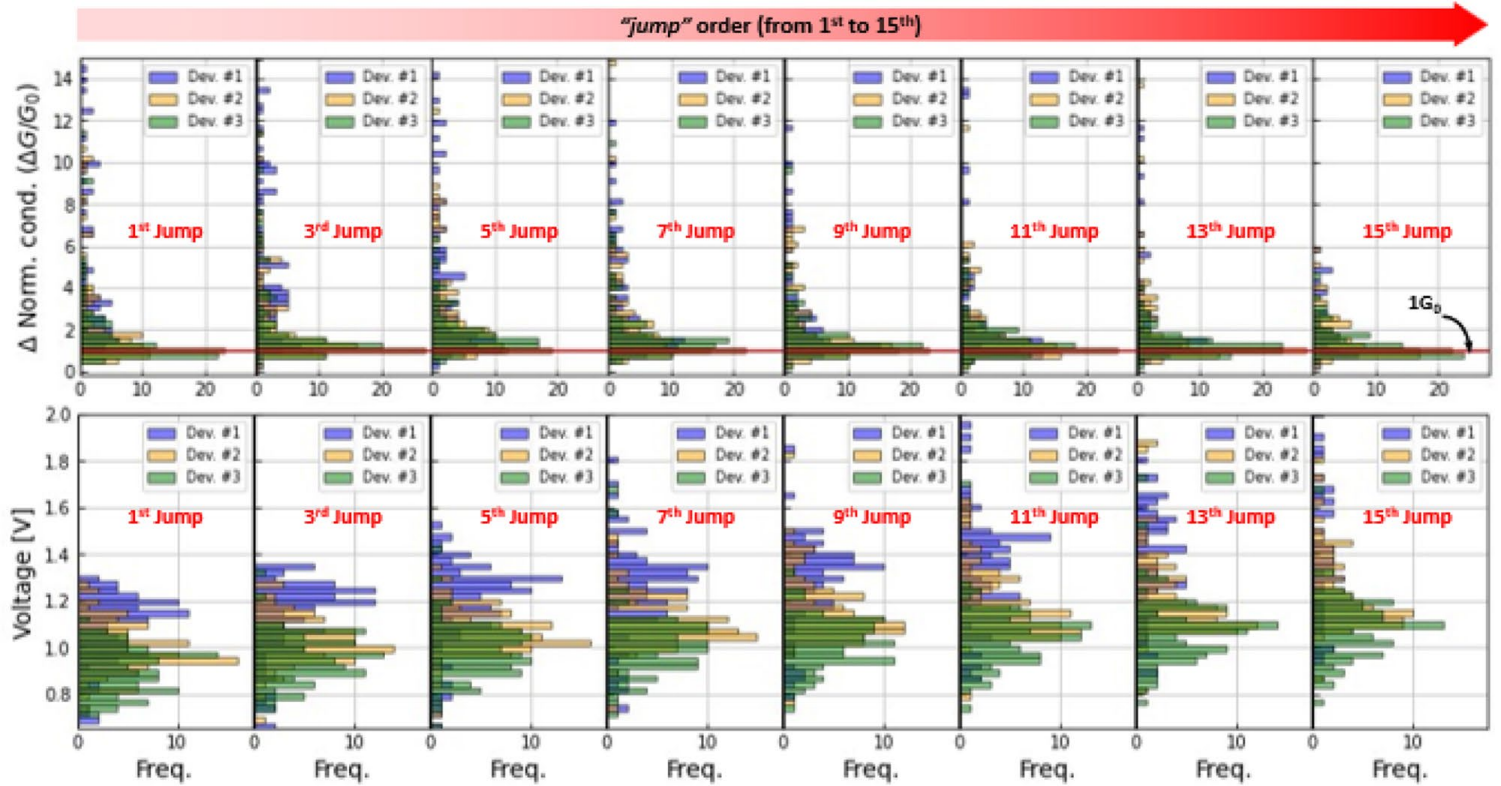
Histograms of the quantum conductance transitions as a function of the transition order (1st jump up to 15th jump) (first row). For three different samples, the transitions occur always around 1 *G*_*0*_. Interestingly, if the ramp rate is high, then the first jumps are roughly higher (4–5 *G*_*0*_). Histograms of the quantum conductance transition voltages as a function of the transition order (1st jump up to 15th jump) (second row). As the ramp rate increases, the transitions occur at lower voltages. But for all cases, there is a shift to higher voltages as the order increases, which is expected.

**Figure 4:**
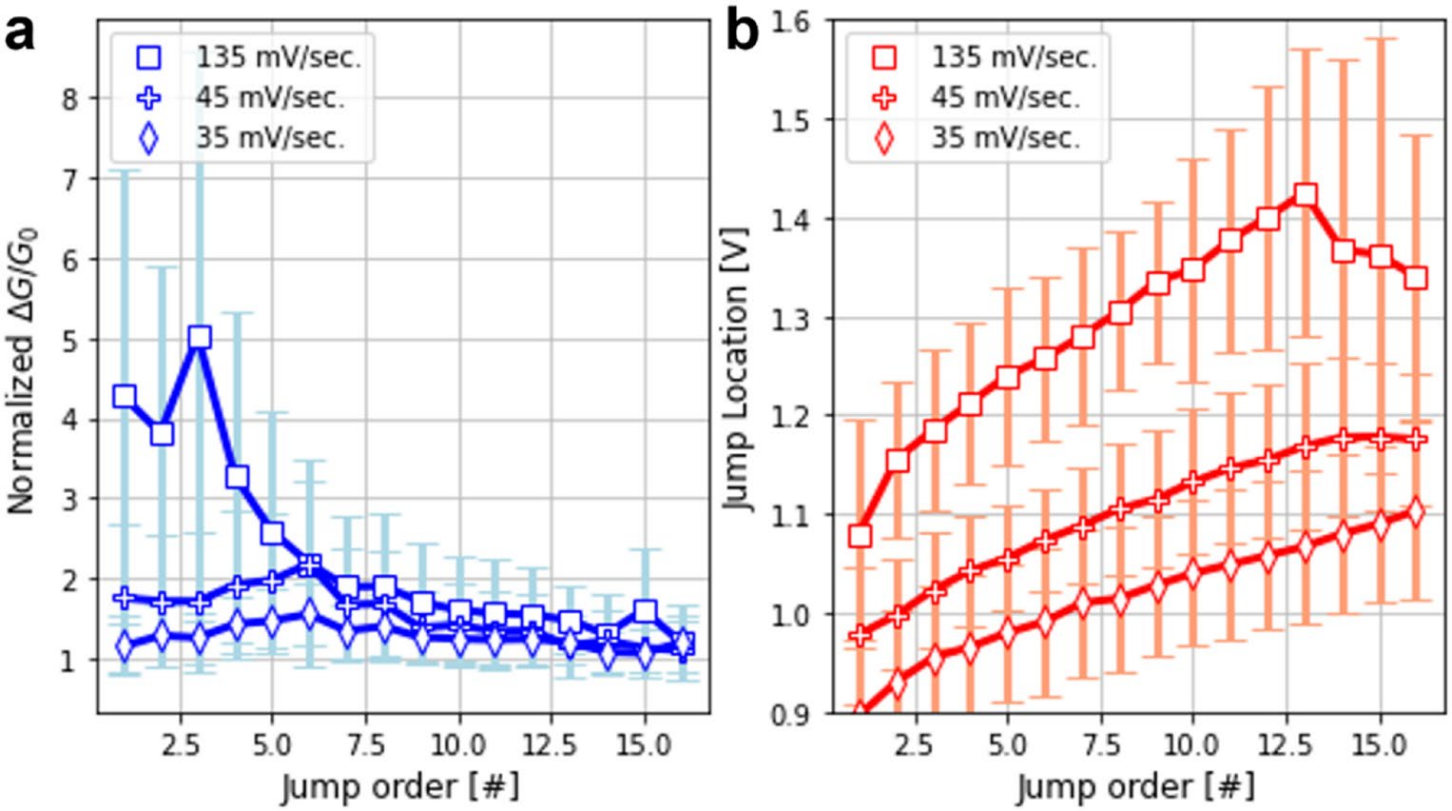
(**a**) Median values for the magnitude of the transitions and for the (**b**) voltage at which each jump occurred. The trends regarding the jump order and the ramp rate are evident. The higher the ramp rate the lower value of the normalized conductance jumps for the low order jumps.

To better understand the dynamics of the transitions, the trends depicted in Fig. 2 were further analyzed by considering the conductance transitions based on their order of occurrence. Here, we describe the transition order as the sequential number of the transition between two consecutive conductance levels, i.e. the first transition in the reset is of first order, the second jump is of second order, and so on. The histograms for the 1st, 2nd, 3rd, 5th, 7th, 9th, 11th, 13th, 15th conductance jumps (see top row in Fig. 3) are separately investigated for the three devices considered in Fig. 2f–k. As the transition order increases, the histograms of all three devices present a peak around ~ 1 *G*_*0*_. Notwithstanding this, the very first transitions show a different behavior, which might be attributed to the different ramp rate used for the voltage sweeps, as for the fastest ramp rate case, the first transitions are normally larger than the rest with a magnitude of ~ 4–5 *G*_*0*_. At the same time, and as expected, the average voltage (see second row in Fig. 3) for the occurrence of a given transition order shifts to higher voltages as the transition order increases. It is worth noticing that the average transition voltage increases with the ramp rate of the voltage sweep, which is in agreement with previously reported experimental observations^38^. These results are summarized in Fig. 4, where the solid lines indicate the median trend and the error bars the dispersion observed for each transition order. Note that the linear trend exhibited by the jump location-jump order curve in most of the investigated range seems to indicate that the filament rupture rate is approximately constant regardless of the actual size of the filament. Last but not least, the correlation plots between *G/G*_*0*_ and *ΔG/G*_*0*_ for each transition order show a progressive evolution from the behavior observed for the 1st transition to that for the 15th transition (See supplementary Fig. 2). The 1st transitions are always observed at high conductance levels and for the samples measured with a voltage ramp rate of 135 mV/s, they show higher values (See supplementary Fig. 2, bottom row, left-most column), suggesting that these transitions are not transitions between quantized states. This is likely a morphology transition in which the consolidated filament becomes more granular and therefore more susceptible of closing independent leakage pathways. As the transition order increases, the magnitude of the transitions decreases to ~ 1 *G*_*0*_ as the absolute conductance levels converge to *G*_*0*_, as described in the previous section. This is ultimately consistent with a single ballistic conducting channel.

**Figure 5:**
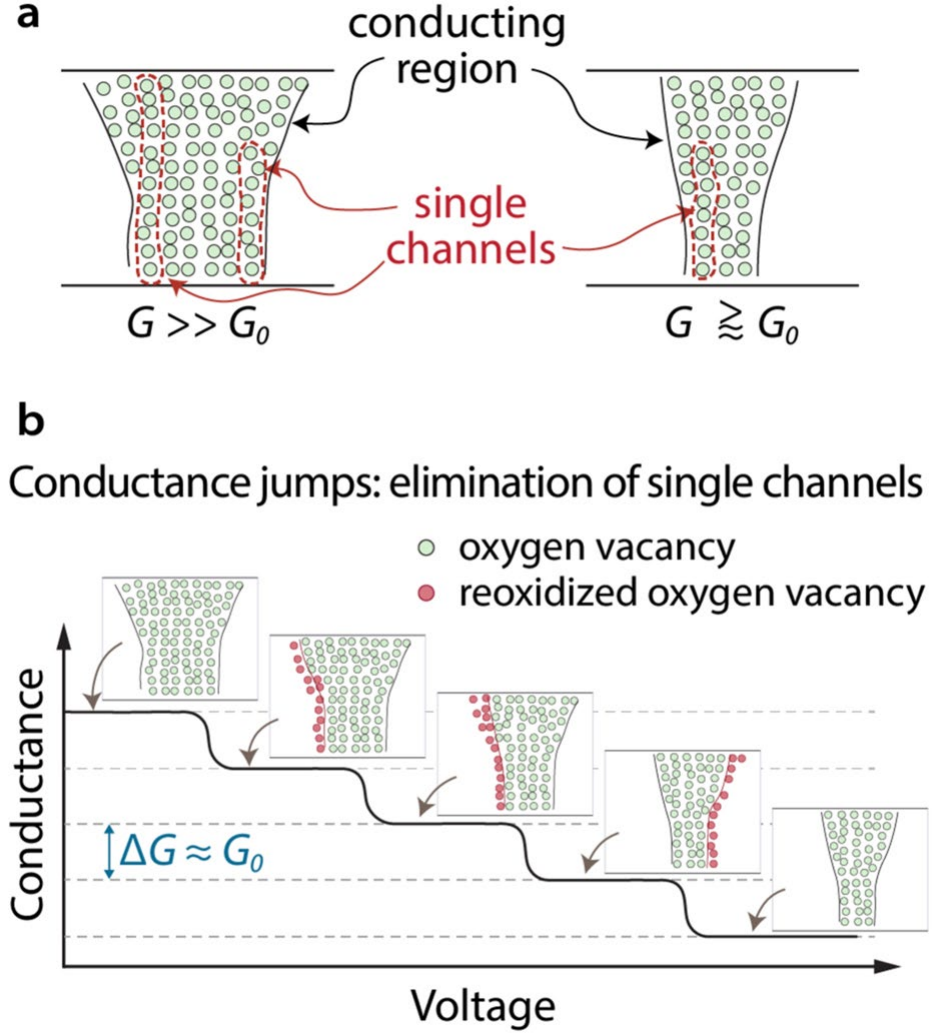
Schematic representation of the conducting region and its morphological changes occurring during reset. (**a**) conducting path morphology in a high and a low conducting state. The conducting region is comprised of several neighboring monomode conducting channels that form a diffusive and a ballistic transport path in the high and low conducting states, respectively. (**b**) Conductance jumps occurring in the reset in multiples of *G*_*0*_ due to elimination of single conduction channels within the conducting region. The conductance becomes quantized when the lateral size of filament is shrunk to a few atoms width.

However, the trends for the slowest voltage ramp rates (35 and 45 mV/s, yellow and green markers in Fig. 3, top row and Supplementary Fig. 2, top and middle row,) to show conductance jumps occurring in multiples of *G*_*0*_ units are remarkable as the initial conductance in the LRS reaches values up to 270 *G*_*0*_, a situation in which conduction cannot be associated with a nanosized ballistic channel. However, the change of conduction still occurs in *G*_*0*_ units, and this is a striking finding that needs to be further elaborated on. A simplified physical picture of the phenomenon is depicted in Fig. 5, with a schematic representation of the conducting region in a highly conductive state and in a state where conduction converges to 1 *G*_*0*_ close to the end of the reset process (Fig. 5a). According to this phenomenological interpretation, the conducting region consists of multiple interacting channels, each with a conductance of about 1 *G*_*0*_ and in close proximity of each other, forming a robust conducting path in the LRS. Then, during reset, this path gets narrower and narrower by the elimination of single channels, inducing a conductance change equal to the number of channels annihilated (see Fig. 5b). Therefore, even though the conduction is apparently diffusive because of the contribution of several channels, in the long run the change of conductance shows quantization effects. Of course, since the conductor in this case is formed from a metal-rich phase in the oxide, i.e., oxygen vacancies, dispersion should be expected around the quantized levels, as it is observed in the experimental data. Since the reset process consists of the elimination of single channels, a transition is expected from diffusive transport to the mesoscopic, ballistic regime below a critical number of remaining channels, and then to tunnelling with the closure of all channels. We expect that the above-described phenomenon can be universally expanded to other oxide-based RRAM devices that show quasi-analog/semi-gradual switching characteristics, including the well-known Ta–O and Hf–O systems^27,31,39^. Also, the fact that the majority of transitions occurs with a magnitude of ~ 1 *G*_*0*_ independent of the yttria layer’s oxygen content suggests that the use of a scavenger layer should also yield similar results.

## Summary

In summary, the quasi-analog nature of the reset process was analyzed in oxygen engineered yttrium oxide-based RRAM devices with special emphasis on the conductance transitions between intermediate resistive states. Remarkably, these transitions are found to occur predominantly with a magnitude equal to the conductance quantum (*G*_*0*_) even at resistance levels far below 12.9 kΩ, i.e., where electron transport is expected to be diffusive. This finding suggests that the changes in conductance are quantized, even if conduction itself is apparently not. As part of this process and with the increment of the transition order, the voltage at which the conductance jumps occur shifts to higher values and a clear convergence of the jump magnitude towards *G*_*0*_ is observed. The associated trends are independent of the ramp rates investigated in this study. To provide an explanation to the observed phenomenon, a plausible interpretation of the conducting filament is presented, wherein the conducting region is formed by multiple monomode ballistic conductors in close vicinity to each other. The transition between two adjacent intermediate conductance levels occurs through the elimination of single channels, therefore inducing changes in multiples of *G*_*0*_. These findings suggest that besides the study of absolute conductance quantization in filamentary-type RRAM devices, the change of conductance should also be investigated as a possible tool for applications, like processing-in-memory and neuromorphic computing architectures, where multilevel data storage and analog switching characteristics are required.

### Supplementary Information

Supplementary Figures.

## Data Availability

The datasets generated during and/or analysed during the current study are available from the corresponding author on reasonable request.
