# Supplementary material for: Revealing the quantum nature of the voltage-induced conductance changes in oxygen engineered yttrium oxide-based RRAM devices
Source: Sci Rep. 2024 Jan 11;14:1122. doi: 10.1038/s41598-023-49924-2 (PMC10784569; doi:10.1038/s41598-023-49924-2)
Supplement: Supplementary file 1 — Supplementary Figures. [file 41598_2023_49924_MOESM1_ESM.pdf]

Supplemental Material for

"Revealing the quantum nature of the voltage-induced conductance changes in oxygen engineered yttrium oxide-based RRAM devices"

F. Aguirre[1*], E. Piros[2*], N. Kaiser[2], T. Vogel[2], S. Petzold[2], J. Gehrunger[3], C. Hochberger[3], T. Oster[4], K. Hofmann[4], J. Suñé[1], E. Miranda[1†] and L. Alff[2†]

Scientific reports, 2023

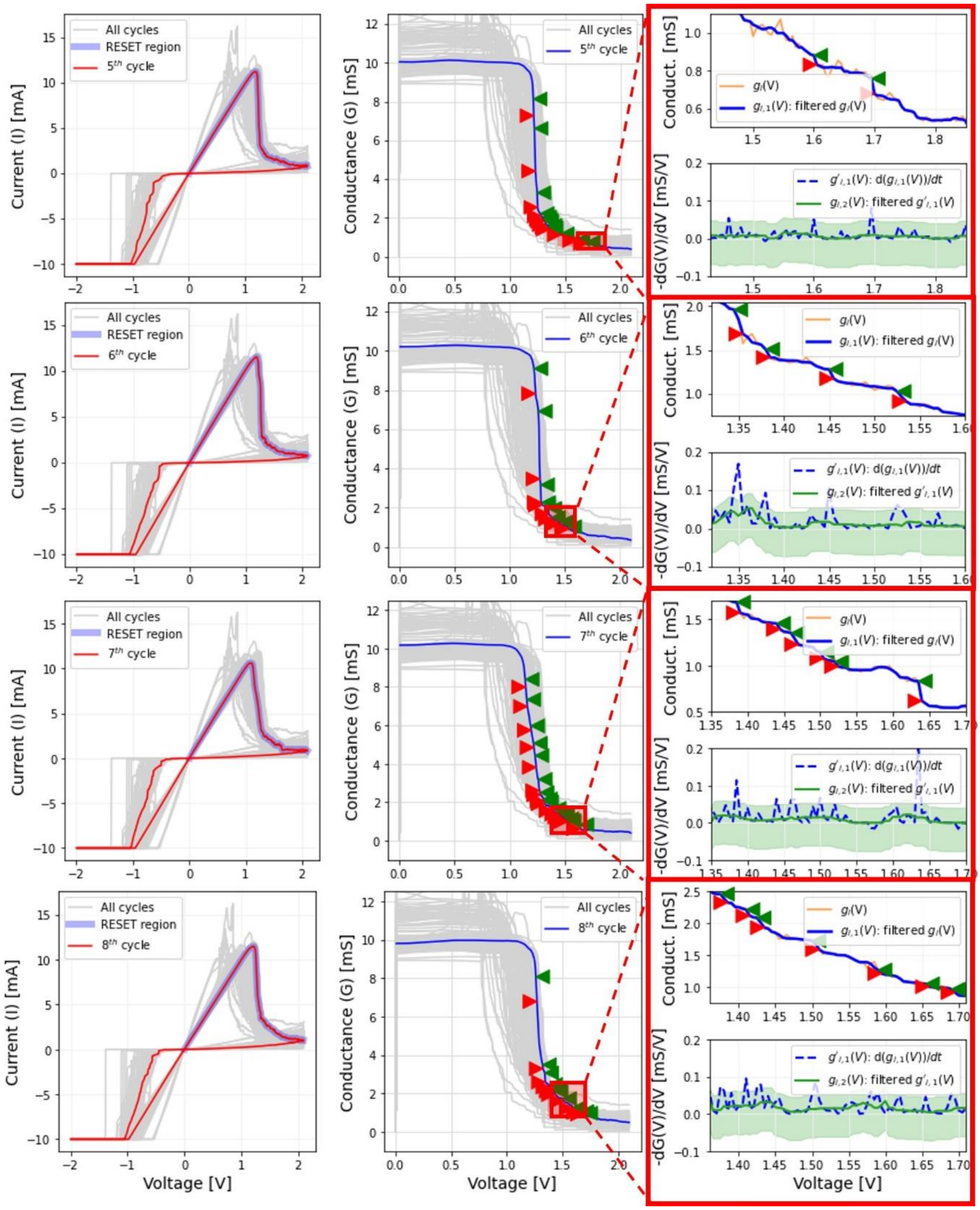

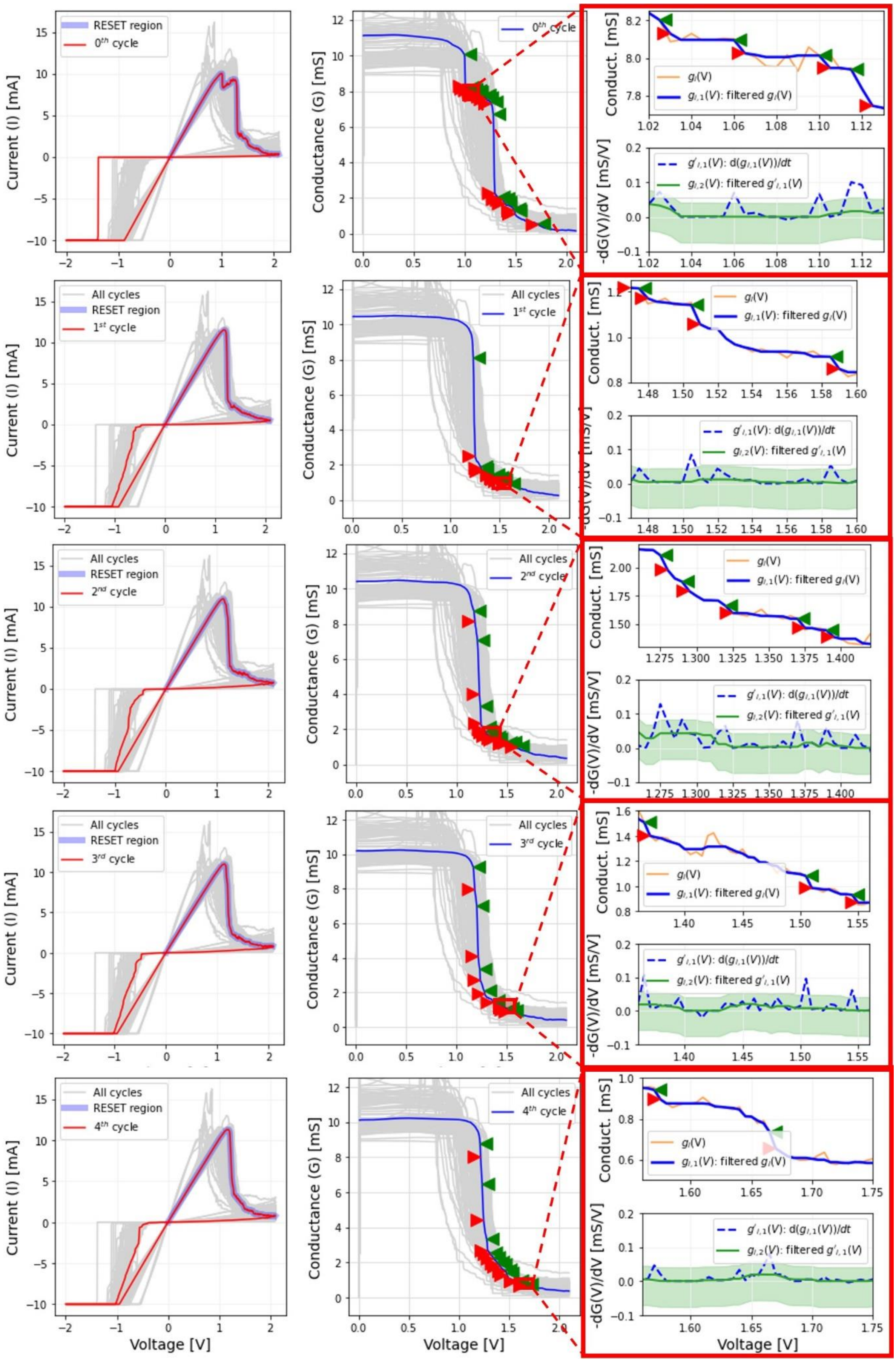

RESET region
0th cycle
All cycles
1st cycle
2nd cycle
3rd cycle
4th cycle
Current (I) [mA]
Conductance (G) [mS]
Conduct. [mS]
-dG(V)/dV [mS/V]
Voltage [V]

Suppl. Figure 1. Exemplary analysis of *I-V* loops in the least oxidized sample (0.1 sccm oxygen flow during growth) with method described in the main text.
right column: selected representative *I-V* curves from 100 dc switching cycles. The region (reset transition) used to extract the information about the transitions is highlighted in blue.
middle column: *G-V* representation of the reset transition calculated by dividing the measured current by the applied voltage. The green and red markers identify the starting and ending point of each jump.
left column: Zoom-in-panel showing a detail of the identified jumps (showing both the raw *G-V* curve and the filtered *G-V* trace (top) and their assessment using the derivative of the signal (bottom)

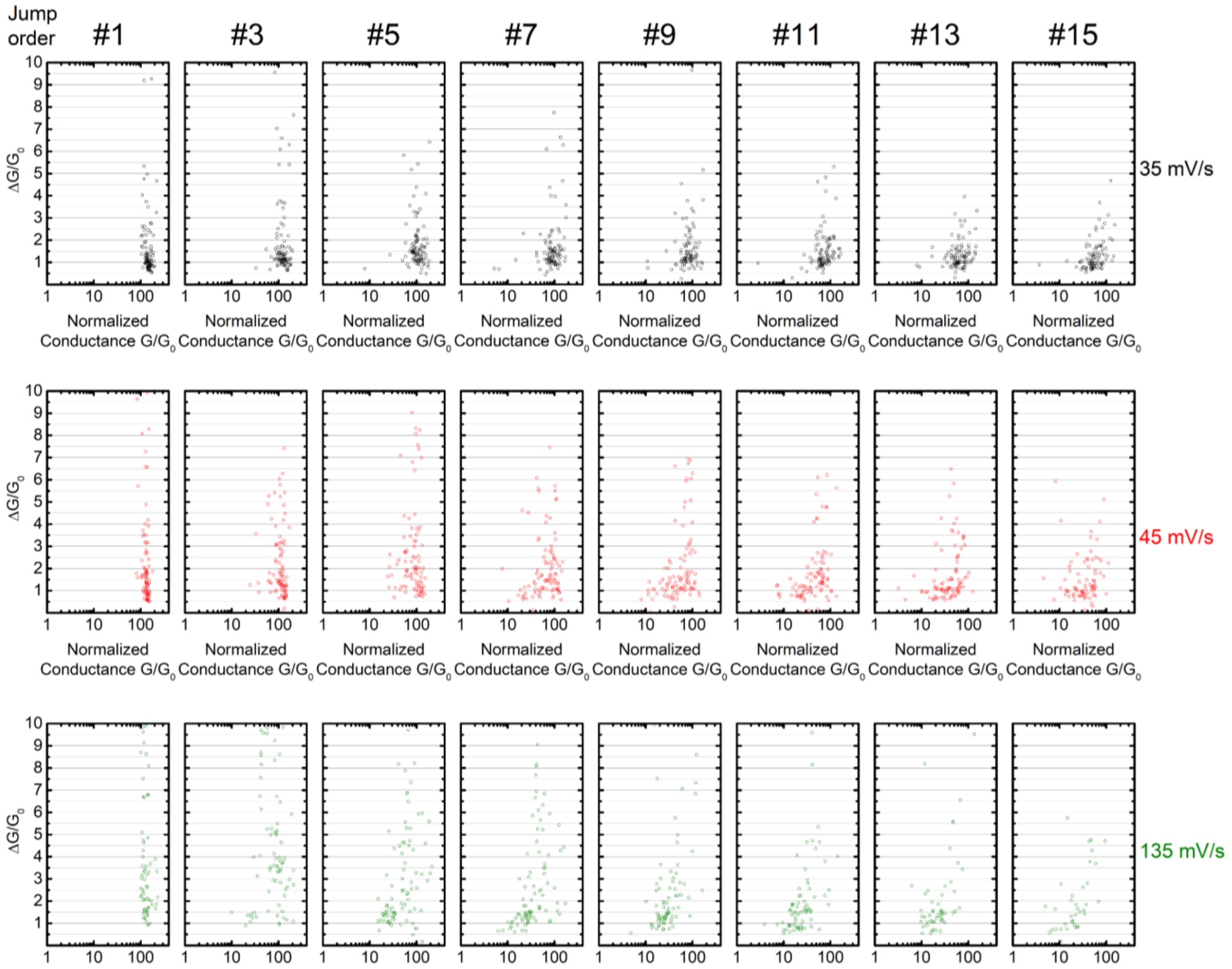

Suppl. Figure 2. Correlation plots between the normalized conductance transitions ($\Delta G/G_0$) and the normalized conductance levels (G/G_0) for the least oxidized sample (0.1 sccm O* flow) for three devices measured with different voltage ramp rates. The conductance jump order increases from 1 to 15 from left to right. For all devices a clustering is visible around $\Delta G/G_0$ = 1 that is more pronounced at low jump orders for the devices with lower ramp rates.
